# Epidemiology of Arterial Hypertension in Kazakhstan: Data from Unified Nationwide Electronic Healthcare System 2014–2019

**DOI:** 10.3390/jcdd9020052

**Published:** 2022-02-05

**Authors:** Sauran Yerdessov, Kainar Kadyrzhanuly, Yesbolat Sakko, Arnur Gusmanov, Gulnur Zhakhina, Dinara Galiyeva, Makhabbat Bekbossynova, Alessandro Salustri, Abduzhappar Gaipov

**Affiliations:** 1 Department of Medicine, Nazarbayev University School of Medicine, Nur-Sultan 010000, Kazakhstan; sauran.yerdessov@nu.edu.kz (S.Y.); kainar.kadyrzhanuly@nu.edu.kz (K.K.); yesbolat.sakko@nu.edu.kz (Y.S.); arnur.gusmanov@nu.edu.kz (A.G.); gulnur.zhakhina@nu.edu.kz (G.Z.); d.galiyeva@nu.edu.kz (D.G.); alessandro.salustri@nu.edu.kz (A.S.); 2 National Research Cardiac Surgery Center, Nur-Sultan 010000, Kazakhstan; mcardio_s@mail.ru

**Keywords:** hypertension, systolic blood pressure, diastolic blood pressure, epidemiology, registry, Kazakhstan

## Abstract

The in-depth epidemiology of hypertension has not been studied in Kazakhstan (KZ) yet. We aimed to investigate the crude; age and sex standardized prevalence, incidence, and all-cause mortality rate among hypertensive patients in Kazakhstan using a large-scale Unified National Electronic Health System (UNEHS) for the period 2014–2019. Hypertension was defined based on the ICD-10 codes (ICD-code: I10; I11; I12; I13). Of 1,908,419 patients, 1,186,706 (62.18%) were females and 721,713 (37.82%) were males. The majority of the patients (56.3%) were ethnic Kazakhs, 26.6% were Russians, and 16.2% were of other ethnicities. In 2014, the crude rates of prevalence, incidence, and mortality were 3661, 1396.1, and 33.1 per 100,000 population, respectively. The overall prevalence, incidence, and mortality rates among hypertension patients had a gradual increase over the period 2014–2019. The sex and age adjusted rates demonstrate the same trend throughout the entire period. We observed 71% higher risk of crude death in males comparing to females (Hazard ratio (HR): 1.71 [95%CI: 1.69–1.72]); Russian and other ethnicities had 1.56-fold (95%CI: 1.54–1.58 and 1.43-fold (95%CI: 1.41–1.45) higher risk of all-cause death compared to Kazakhs, and the elderly group had the highest risk of death (Hazard ratio (HR): 35.68 [95%CI: 28.11–45.31]) comparing to the younger generation, which remained significant after adjustment to age and sex. Overall, these findings show statistically significant lower survival probability in male patients compared to female, in older patients compared to younger ones, and in patients of Russian and other ethnicities compared to Kazakh.

## 1. Introduction

Approximately 1.13 billion people of the worldwide population have hypertension, the majority (approximately 66%) live in low and middle-income countries [1]. The prevalence of arterial hypertension (AH) in Russia has increased in recent years from 2008 to 2014 [2], while several studies have demonstrated a linear relationship between the level of blood pressure (BP) and the frequency of its complications [3].

It is estimated that hypertension kills 7.5 million individuals each year [4]. Based on the Global Burden of Disease study, around 3.5 billion adults worldwide had SBP of 110–115 mmHg in 2015, a level that is connected with increased risk of ischemic heart disease (IHD), stroke, and kidney disease [5]. As a continuation of this trend, with the updated definition of hypertension in 2017, the USA had an increase in the prevalence of hypertensive disease from 32.0% to 45.4% [6].

Data from epidemiological studies by Alikhanova et al. shows that the leading morbidities of the adult population are attributed to diseases of the circulatory system, which are manifested by an increase in blood pressure (7801.4 cases per 100 thousand adults) [7].

In Kazakhstan, there is an increase in the incidence of cardiovascular diseases consistent with the worldwide trend, which can be explained by an increase in the quality of screening examinations, improved detection (daily blood pressure monitoring, etc.), treatment, and also by a decrease in the availability and quality of medical care [8,9,10,11].

However, the data of K. K. Davletov et al. show that in recent years, in the Republic of Kazakhstan, there has been a significant decrease in the total mortality rate from cardiovascular disease (CVD)—from 535.5 per 100 thousand population in 2005 to 316.0 per 100 thousand population in 2011, while the incidence of CVD does not decrease and even increases [12,13]. The problem is that the indicators of the incidence of CVD in Kazakhstan do not reflect the true new cases, since they are estimated mainly by the population’s access to medical care, and the mortality rates from CVD are not standardized by age, as is customary in international cardiology and epidemiology [14,15,16]. Despite the high mortality and the importance of hypertension as a cardiovascular risk factor, there is not much reliable information on hypertension within the Central Asian countries of the former Soviet Union [17].

To date, there was no epidemiologic study that provided large representative data of hypertensive patients in Kazakhstan. Taking into account this knowledge gap, we aimed to study descriptive epidemiology (prevalence, incidence, and mortality rate) of hypertension in Kazakhstan using unified nationwide electronic healthcare records for the period 2014–2019. We also aimed to analyze the association between population demographics and all-cause mortality.

## 2. Materials and Methods

### 2.1. Study Population

**Figure 1:**
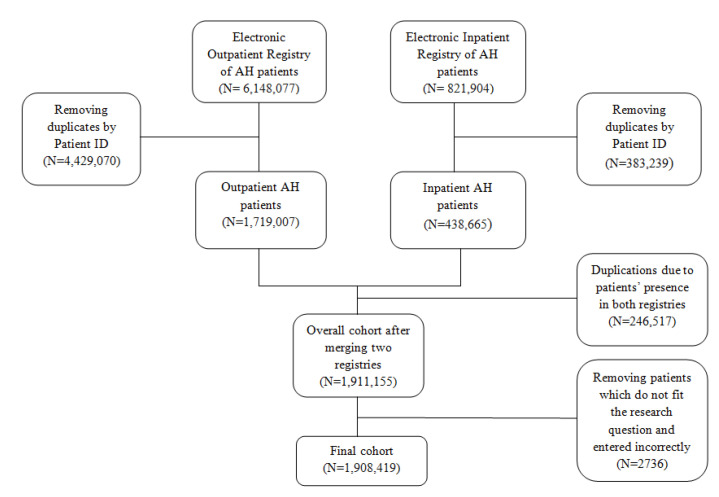
The flowchart diagram.

This is a retrospective cohort study of the population diagnosed with arterial hypertension documented in outpatient and/or inpatient setting in Kazakhstan. The data were extracted from the Unified National Electronic Health System (UNEHS), which is a nationwide electronic healthcare system, obtaining health records from medical organizations and their provision at the national level. The initial cohort consisted of 821,904 entries from electronic registry of inpatient data (ERID) and 6,148,077 observations from electronic registry of outpatient data (EROD). A total raw data of 6,969,981 patients with AH existing in UNEHS between 2014 and 2019 were extracted from both ERID and EROD using ICD-10 codes I10 (essential (primary) hypertension). After combining the two databases, duplicate records with the same population registry number (RPN ID) were removed. Finally, 1,908,419 out of 6,969,981 patients had been included for further statistical evaluation (Figure 1).

The diagnosis of hypertension was based on ICD-10 codes (ICD-10 code: I10; I11; I12; I13).

### 2.2. Exposures and Covariates

Patients’ personal data included RPN ID, date of birth, date of death, age, gender, ethnicity, residency, date of admission, date of discharge, ICD-10 code. The date of the first appointment might be registered to the UNEHS reflectively from past impermanent electronic frameworks and old libraries in case they were entered before 2014. Data about date of birth and death were retrieved through linkage with the Population Registry through population registry number (RPN number). The information on comorbidities such as diabetes, stroke, and chronic kidney disease was obtained from the same database. Age was categorized into seven groups (18–24, 25–34, 35–44, 45–54, 55–64, 65–74, and above 75 y.o.), ethnicity was categorized as Kazakhs, Russians, and others (included Ukrainians, Uyghurs, Uzbeks, Koreans, etc.) and the residency was categorized as Rural and Urban.

### 2.3. Outcome Assessment

The prevalence, incidence, and all-cause mortality of AH patients were assessed. For each year, the prevalence was calculated by dividing all alive patients diagnosed with hypertension by the average total population. Similarly, incidence and mortality were calculated by dividing the number of new diagnosis and deaths, respectively, by the average total population size during the year. The population parameters of Kazakhstan were obtained from the Statistics Committee [18]. The beginning of the follow-up period was the date of the first confirmation of the diagnosis (which might be before 2014) based on the first date of registry in the EROD of the first admission to the hospital with hypertension (first registration in the ERID), and patients were monitored until death or other censoring events or the end of the follow-up period (December 31th, 2019).

### 2.4. Statistical Analysis

Data were represented as descriptive, where absolute values and percentages for categorical variables were calculated. In the bivariate analysis, independent *t*-tests and χ^2^ tests were used to examine the relationship between numeric and categorical variables, respectively.

Trends on prevalence, incidence, and mortality rates for 2014–2019 were represented as crude; sex and age adjusted values using the whole population in 2014 as the standard population and the following seven age groups: 18 to 24, 25 to 34, 35 to 44, 45 to 54, 55 to 64, 65 to 74, and 75 years or older [18]. Prevalence, incidence, and mortality rates within the hypertension population are presented as per 100,000 populations. Additionally, the mortality rate within the hypertension population is presented as per 1000 patient years (/1000PY) in different groups.

To estimate the risk of all-cause mortality among hypertensive patients, Cox proportional hazards regression analysis was used to obtain unadjusted and adjusted hazard ratios and their corresponding confidence intervals. The adjustments were made for age and sex, based on theoretical considerations and after checking its assumptions. *p* values are two-sided and reported as significant at <0.05 for all analyses. Data management and statistical analysis were performed using STATA 16.1 MP2 Version (STATA Corporation, College Station, TX, USA).

## 3. Results

### 3.1. Demographic Data

The demographic characteristics of the cohort are presented in Table 1. A total number of 1,908,419 patients (37.82% men and 62.18% women) were included in the analyses. Overall, the mean age (±SD) was 59.2 ± 11.7 years. Of patients, 56.3% were Kazakhs, 26.6% Russians, and 16.2% were listed as other ethnicities. The age group 55–64 years had the highest prevalence of hypertension. Fifty-three percent of the cohort were urban residents. Diabetes, stroke, and CKD were present in 14.5%, 4,6%, and 0.42% of patients, respectively.

### 3.2. Prevalence and Incidence

The Figures S1–S3 (Supplementary Material) present absolute, crude, and adjusted (sex and age) rates and interpret the data gathered on the incidence for men and women, place of residence of the patients (urban and rural) in the Republic of Kazakhstan per 100,000 inhabitants. From 2014 to 2019, there is a consistent growth in the number of cases, especially among women living in the city. It reached its peak in both figures (Figures S2 and S3) in 2016, showing a crude rate of 2835.1 per 100,000 population for female and 2010 per 100,000 population for male, while the urban rate of 2418.6 per 100,000 population was also high with a lower rate for rural 1685.3 per 100,000 population.

**Figure 2:**
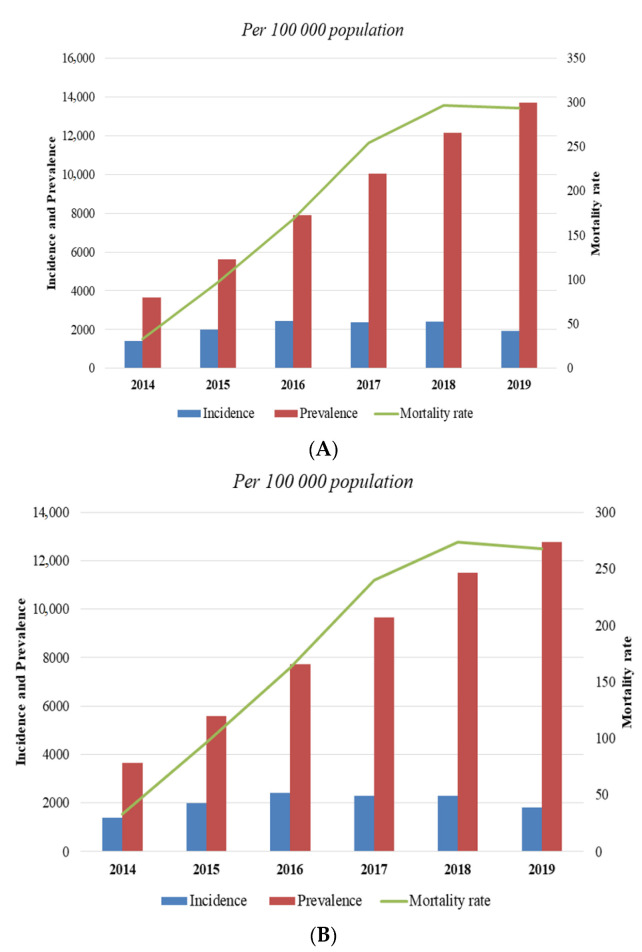
Crude (**A**) and age and sex standardized (**B**) prevalence, incidence, and mortality rates of hypertension in Kazakhstan for 2014–2019.

Comparing the indicators for the Republic of Kazakhstan, the crude prevalence increased from 3661 to 13,693.8 per 100,000 population, similar to the incidence rate that increased from 1396.1 to 1927.5 per 100,000 population. A similar trend is observed in the adjusted rates, showing an increase in the prevalence from 3661 to 12,775.2 per 100,000 population and incidence from 1396.1 to 1798.7 per 100,000 population. Moreover, the number of mortality rates increased from 33.1 to 293.7 per 100,000 population, as well as adjusted mortality rates from 33.1 to 267.6 per 100,000 population (Figure 2).

### 3.3. All-Cause Mortality

During the median 3.33 years [IQR: 1.72–5.39] of the follow-up period, there were 142,956 deaths (7.49% of the cohort). There is a rise in the crude mortality rate between 2014 and 2019 (Figure S4 in Supplementary Material), which shows the same trend as age and sex adjusted mortality rate. From Table 1, the crude mortality rate per thousand patient years (/1000PY) was higher in males compared to females (24.89/1000PY [95%CI: 24.70–25.08] vs. 14.79/1000PY [95%CI: 14.69–14.90]), in those of Russian ethnicity compared to Kazakhs (23.5/1000PY [95%CI: 23.30–23.71] vs. 14.91/1000PY [95%CI: 14.81–15.04]) and in older patients compared to younger ones. Finally, chronic kidney disease has a higher mortality rate compared to diabetes and stroke ([63.47/1000PY [95%CI: 60.81–66.24] vs. 23.3/1000PY [95%CI: 23.04–23.57] and 55.01/1000PY [95%CI: 54.25–55.76], respectively).

**Figure 3:**
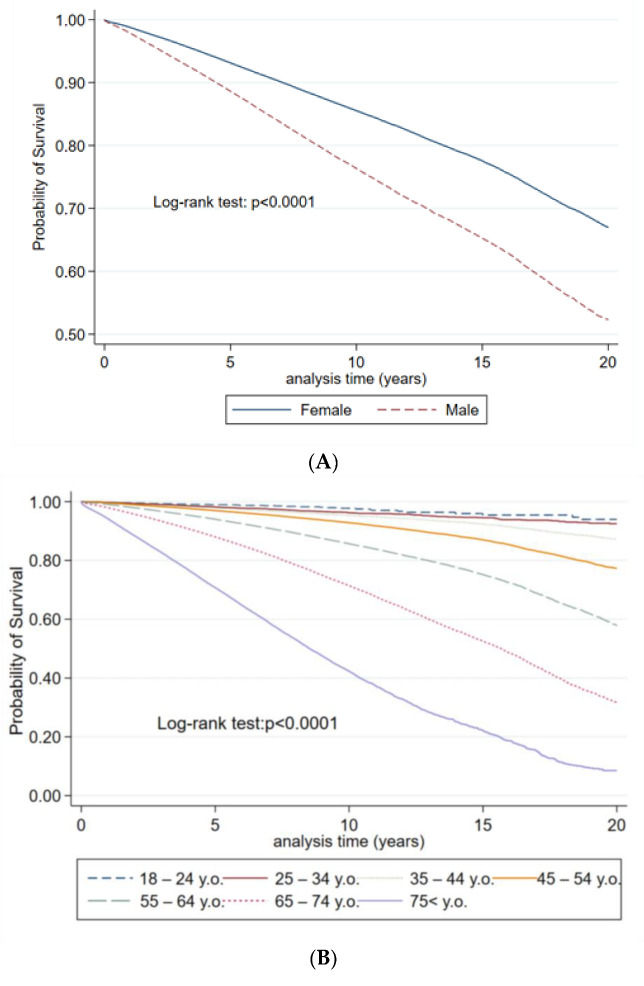

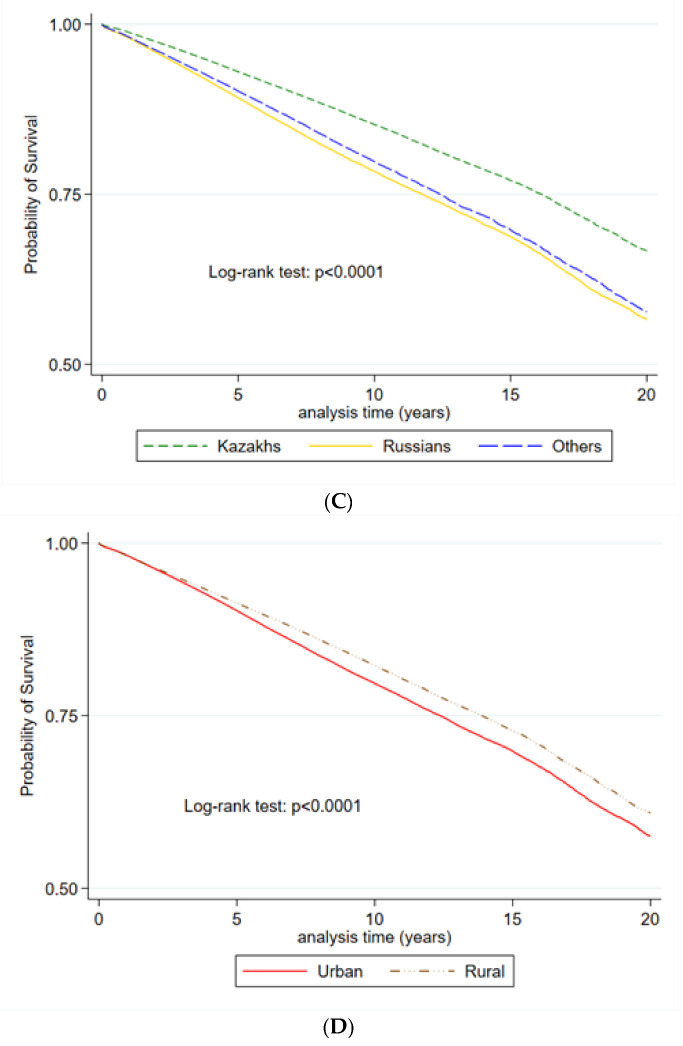
Unadjusted Kaplan–Meyer graphs of hypertension in Kazakhstan by gender (**A**), age category (**B**), nationality (**C**), and place of residence (**D**).

Males had a higher risk of death (HR = 1.71, [95%CI: 1.69–1.72], *p* < 0.001), from arterial hypertension compared to females, however, patients living in rural areas show the opposite result, with 12% lower risk of death (HR = 0.88, [95%CI: 0.88–0.89], *p* < 0.001), compared to patients residing in urban areas. The risk of death increases with age—people in the age group 18–24 is the reference group. Finally, by ethnicity groups, Russians and others ((HR = 1.56, [95%CI: 1.54–1.58] and HR = 1.43, [95%CI: 1.41–1.45], *p* < 0.001), respectively) have a higher risk comparing to the Kazakhs (Figure 3). Patients who had diabetes, stroke, and CKD demonstrated 1.32-fold, 3.31-fold, and 3.49-fold higher risk of crude mortality, respectively, with similar trend after adjustments (Table 2).

However, from Cox proportional hazards regression analysis adjusted for age and sex, risk of death from hypertension slightly decreased in Russians (HR = 1.18, [95%CI: 1.17–1.19, *p* < 0.001] and others (HR = 1.15, [95%CI: 1.13–1.17], *p*-value < 0.001), while increased in patients from rural area (HR = 1.06, [95%CI: 1.05–1.07], *p* < 0.001). As for comorbidities, the risk of death decreased (HR = 2.69 [95%CI: 2.65–2.73], *p*-value < 0.001) in patients who have stroke compared with those who did not, while diabetes and chronic kidney disease showed an opposite trend (HR = 1.35 [95%CI: 1.33–1.36], *p*-value < 0.001 and HR = 4.54 [95%CI: 4.35–4.74], *p*-value < 0.001, respectively) (Table 2). The detailed information on Cox regression model adjusted for all variables is given in Table S1 (Supplementary Material).

## 4. Discussion

This is the first research in Central Asia on the epidemiology of AH using a large-scale healthcare database from Unified National Electronic Health System. The results showed an overall increase in prevalence, incidence, and mortality rates of hypertension from 2014 to 2019. Interestingly, after adjustment for age and gender, there is a statistically significant higher survival probability of ethnic Russians or other compared to previous unadjusted results, and a decrease in survival probability of rural compared to urban places.

Bermagambetova et al. results showed that both in the city and in the rural areas there is almost the same degree of hypertension incidence, which can be compared with international data. However, our analysis indicates that the numbers of hypertensive patients located in the city was higher compared to rural places, which suggests that living in urban areas is a risk factor for arterial hypertension [19,20,21]. Low physical activity of the population, changes in consumer behavior, and increased stress are the consequences of urbanization [20]. As a result, changing living environment, lifestyle, gaining weight, and weight problems may lead patients to hypertension [22].

The relationship between increased blood pressure, age, and gender has been known since a long time. Our findings demonstrate an increase in the incidence of hypertension as patients gets older, especially in women. Similar findings were reached in studies that looked at the link between a patient’s gender, age, and incidence rate. As an example, in the Framingham Heart Study, it was observed that after the age of 60, the incidence in women becomes higher than in men. This may be due to the selective removal of men from the risk group since at the initial level they have higher rates of hypertension and a lower survival rate because of cardiovascular deaths [23]. Additionally, menopause could play a role as it is associated with hormones-related endothelial dysfunction [24]. Furthermore, in works of O’rourke and Hashimoto by aging, central arteries can be stiffed and dilated due to repetitive pulsations, causing rise of aortic systolic pressure [25]. The prevalence of hypertension is increasing steadily in developing countries, including Kazakhstan. Our results are consistent with previous information, which showed an increase in the incidence of hypertension in the Republic of Kazakhstan from 1147.89 to 1970.18 per 10,000 inhabitants between 1997 and 2009. The prevalence of hypertension in Kazakhstan, according to various sources, ranges from 15 to 28%, and both in the cities and in the villages, there is almost the same incidence, which can be compared with international data [19].

At the same time, mortality rates related to hypertension rose as well and currently rank first among the causes of death. Approximately 40% of the deaths are observed during active working age (20–64 years), of which 64% in the male population, which is in line with previous studies showing higher risk of death (approximately 39%) in the active working age group. [26]. According to the World Health Organization (WHO), premature mortality from CVD in Kazakhstan ranks second amongst the European region countries [4,27].

AH is a common problem to the Kazakhstan public health sector as in the majority of the world’s countries and poses a significant threat to it, as according to the official statistics, the prevalence of AH affects 24.3% of the adult people in Kazakhstan [26,28]. Between 2009 and 2013, this prevalence has considerably grown from 10,777.7 to 13,391.6 per 100,000 inhabitants [28,29]. While we do not have up-to-date information on the prevalence of hypertension, our findings appear to be consistent with prior research that shows a rise in number of prevalent cases.

There are several limitations in this study that deserve mentioning. The real prevalence of hypertension could be underestimated due to lack of access to healthcare, misclassification of the cases of hypertension, and miscoding of hypertension. We were not able to define a cause-specific mortality data and associations were accounted only for all-cause mortality. Although the impact of few comorbidities such as diabetes, stroke, and chronic kidney disease was assessed, other important confounders were missing, which could have a potential impact on the mortality.

As there were an update in the definition of hypertension in adults by the American Heart Association (AHA) and the International Society of Hypertension (ISH) according to the indicators of systolic blood pressure (SBP) and diastolic blood pressure (DBP) in 2017, the coding of hypertension might be misclassified during the study period [30,31,32].

## 5. Conclusions

This is the first retrospective study in Kazakhstan to address the prevalence, incidence, and all-cause mortality rate of AH patients utilizing the large-scale nationwide healthcare records over a six-year period between 2014 and 2019. The results showed an increase in the prevalence, incidence, and mortality rate of AH patients. We observed a lower survival probability in male patients compared to females, in older patients compared to younger ones, and in patients of Russian and other ethnicities compared to Kazakh. Future studies should concentrate on the development of actions to improve measures of primary and secondary prevention of AH, taking into account the main risk factors for their development.

## Figures and Tables

**Table 1:** Baseline characteristics of patients, proportion of gender and crude mortality rate per 1000 patients’ year (*n* = 1,908,419) for 2014–2019.

Variables	Overall 1,908,419	Male 721,713 (37.82%)	Female 1,186,706 (62.18%)	*p*-Value	Mortality Rate per 1000 Person Year [95%CI]
Male					24.89 [24.70–25.08]
Female					14.79 [14.69–14.90]
Age groups					
18–24	6356 (0.33%)	4136 (65.07%)	2220 (34.93%)	<0.001	2.6 [2.05–3.30]
25–34	28,595 (1.5%)	13,054 (45.65%)	15,541 (54.35%)		3.78 [3.46–4.12]
35–44	153,030 (8.0%)	65,170 (42.59%)	87,860 (57.41%)		4.77 [4.61–4.93]
45–54	468,768 (24.6%)	187,259 (39.95%)	281,509 (60.05%)		7.11 [6.99–7.22]
55–64	659,311 (34.5%)	257,471 (39.05%)	401,840 (60.95%)		13.56 [13.42–13.69]
65–74	380,556 (19.9%)	132,637 (34.85%)	247,919 (65.15%)		27.99 [27.71–28.26]
75<	211,803 (11.1%)	61,986 (29.27%)	149,817 (70.73%)		69.8 [69.17–70.43]
Ethnicity					
Kazakh	1,074,434 (56.3%)	440,206 (40.97%)	634,228 (59.03%)	<0.001	14.91 [14.81–15.04]
Russian	507,709 (26.6%)	159,803 (31.48%)	347,906 (68.52%)		23.5 [23.30–23.71]
Other	309,436 (16.2%)	115,504 (37.33%)	193,932 (62.67%)		21.37 [21.12–21.64]
Missing	16,840 (0.9%)	6200 (36.82%)	10,640 (63.18%)		NA
Resident					
Urban	1,010,433 (53.0%)	369,893 (36.61%)	640,540 (63.39%)	<0.001	21.13 [20.99–21.27]
Rural	592,071 (31.0%)	239,685 (40.48%)	352,386 (59.52%)		19.13 [18.96–19.29]
Missing	305,915 (16.0%)	112,135 (36.66%)	193,780 (63.34%)		NA
Comorbidities					
Diabetes	277,450 (14.5%)	89,055 (32.1%)	188,377 (67.9%)	<0.001	23.3 [23.04–23.57]
Stroke	87,621 (4.6%)	44,823 (51.16%)	42,798 (48.84%)	<0.001	55.01 [54.25–55.76]
Chronic Kidney Disease	8035 (0.42%)	4291 (53.4%)	3744 (46.6%)	<0.001	63.47 [60.81–66.24]

**Table 2:** Multivariable Cox proportional hazards regression analysis with unadjusted and adjusted values for 2014–2019.

Variables	Unadjusted (95%CI)	*p*-Value	Adjusted for Sex and Age (95%CI)	*p*-Value
Gender *				
Women	Ref.		Ref.	
Men	1.71 [1.69–1.72]	*p* < 0.001	1.98 [1.96–2.00]	*p* < 0.001
Age groups **				
18–24	Ref.		Ref.	
25–34	1.41 [1.09–1.81]	*p* < 0.001	1.59 [1.23–2.05]	*p* < 0.001
35–44	1.95 [1.54–2.49]		2.29 [1.81–2.92]	
45–54	3.13 [2.46–3.97]		3.73 [2.94–4.73]	
55–64	6.34 [4.99–8.04]		7.59 [5.98–9.64]	
65–74	13.38 [10.54–16.98]		16.52 [13.01–20.97]	
75<	35.68 [28.11–45.31]		45.66 [35.97–57.97]	
Ethnicity				
Kazakh	Ref.		Ref.	
Russian	1.56 [1.54–1.58]	*p* < 0.001	1.18 [1.17–1.19]	*p* < 0.001
Other	1.43 [1.41–1.45]		1.15 [1.13–1.17]	
Place of residence				
Urban	Ref.		Ref.	
Rural	0.88 [0.88–0.89]	*p* < 0.001	1.06 [1.05–1.07]	*p* < 0.001
Comorbidities				
Diabetes	1.32 [1.30–1.34]	*p* < 0.001	1.35 [1.33–1.36]	*p* < 0.001
Stroke	3.31 [3.26–3.36]		2.69 [2.65–2.73]	
Chronic Kidney Disease	3.49 [3.35–3.65]		4.54 [4.35–4.74]	

* The variable “gender” was adjusted for age groups. ** The variable “age group” was adjusted for gender.

## Data Availability

The data that support the findings of this study are available from Republican Center for Electronic Health of the Ministry of Health of the Republic of Kazakhstan, but restrictions apply to the availability of these data, which were used under the contract-agreement for the current study, and so are not publicly available. Data are however available from the authors upon reasonable request and with permission of Ministry of Health of the Republic of Kazakhstan.

## Supplementary Materials

The following are available online, Figure S1: Absolute numbers of new case, prevalent cases and deaths of hypertension in Kazakhstan for 2014–2019 years; Figure S2: Crude (A) and age standardized incidence (B) rates of hypertension for men and women; Figure S3: Crude incidence rate of hypertension for urban and rural population; Figure S4: Crude (A) and age & sex standardized (B) all-cause mortality rates of hypertension patients in Kazakhstan; Table S1: Multivariable Cox proportional hazards regression analysis with unadjusted and adjusted values for 2014–2019 years.
