# Supplementary material for: Epidemiology of Arterial Hypertension in Kazakhstan: Data from Unified Nationwide Electronic Healthcare System 2014–2019
Source: J Cardiovasc Dev Dis. 2022 Feb 5;9(2):52. doi: 10.3390/jcdd9020052 (PMC8876304; doi:10.3390/jcdd9020052)
Supplement: Supplementary file 1 [file jcdd-09-00052-s001.zip › jcdd-1518946-supplementary.pdf]

Figure S1. *Absolute numbers of new case, prevalent cases and deaths of hypertension in Kazakhstan for 2014 – 2019 years.*

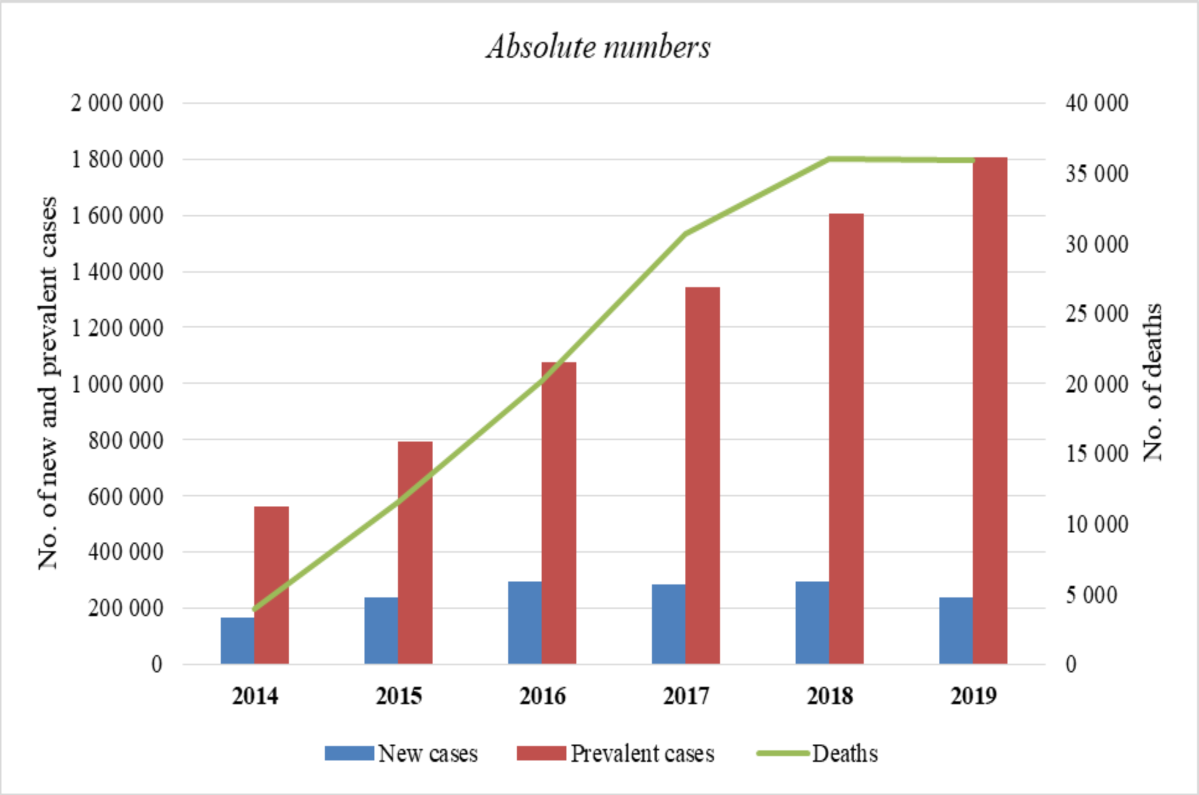

Figure S2. *Crude (A) and age standardized incidence (B) rates of hypertension for men and women.*

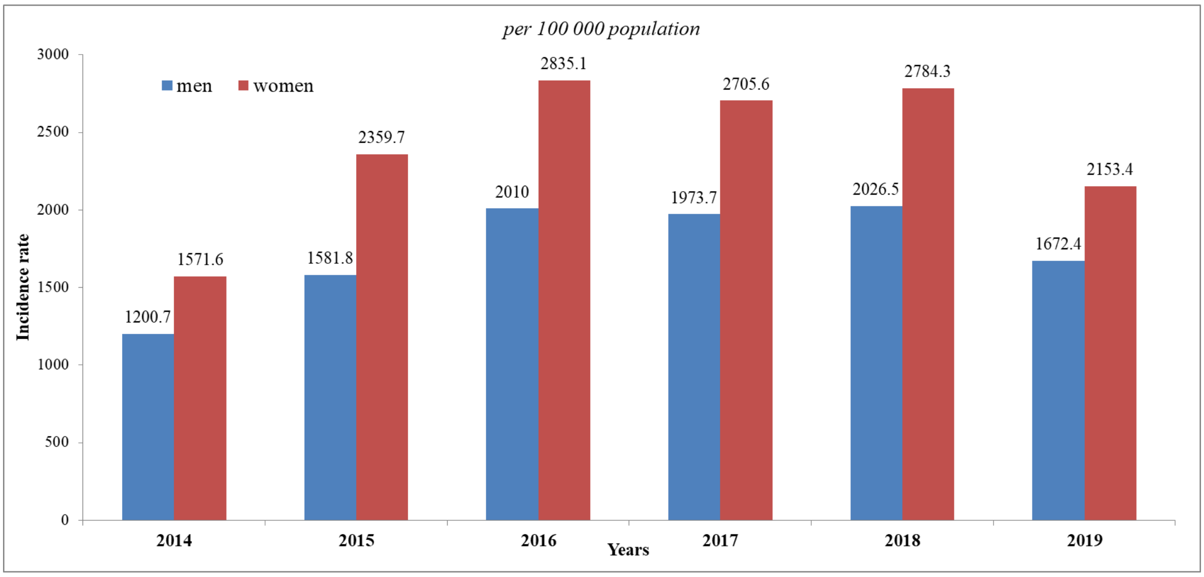

A.

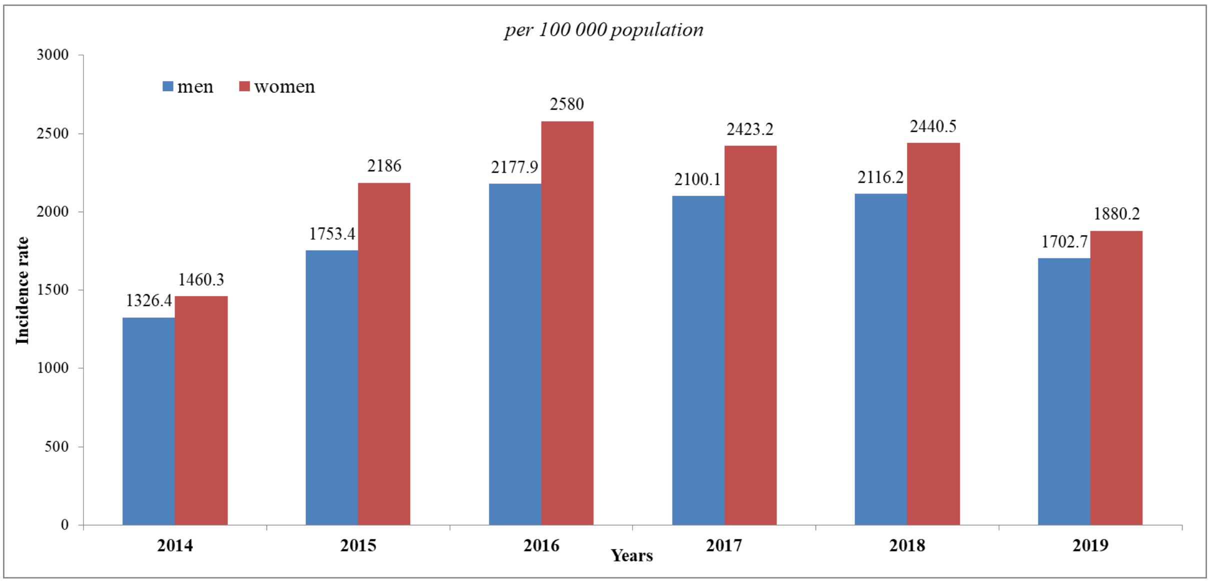

B.

Figure S3. *Crude incidence rate of hypertension for urban and rural population.*

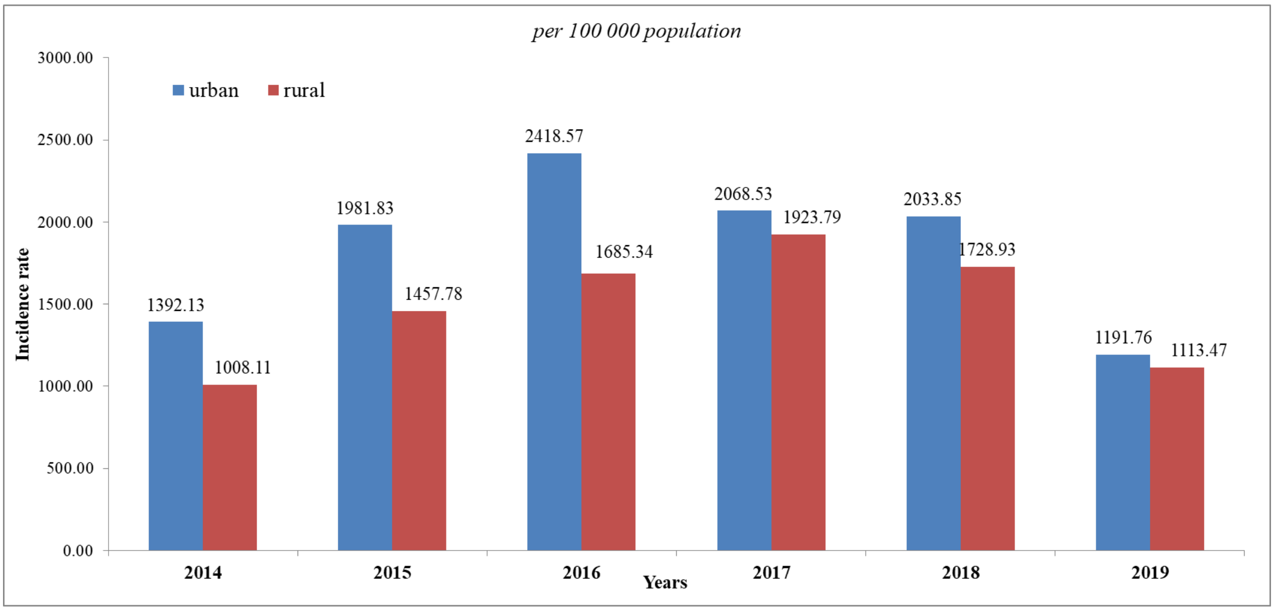

Figure S4. *Crude (A) and age & sex standardized (B) all-cause mortality rates of hypertension patients in Kazakhstan.*

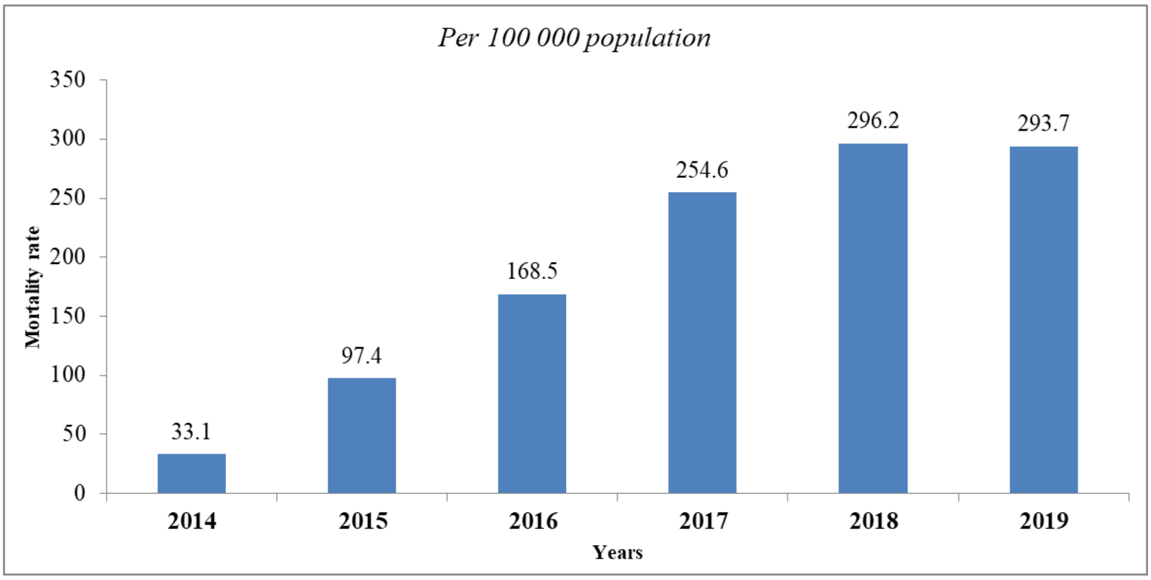

A.

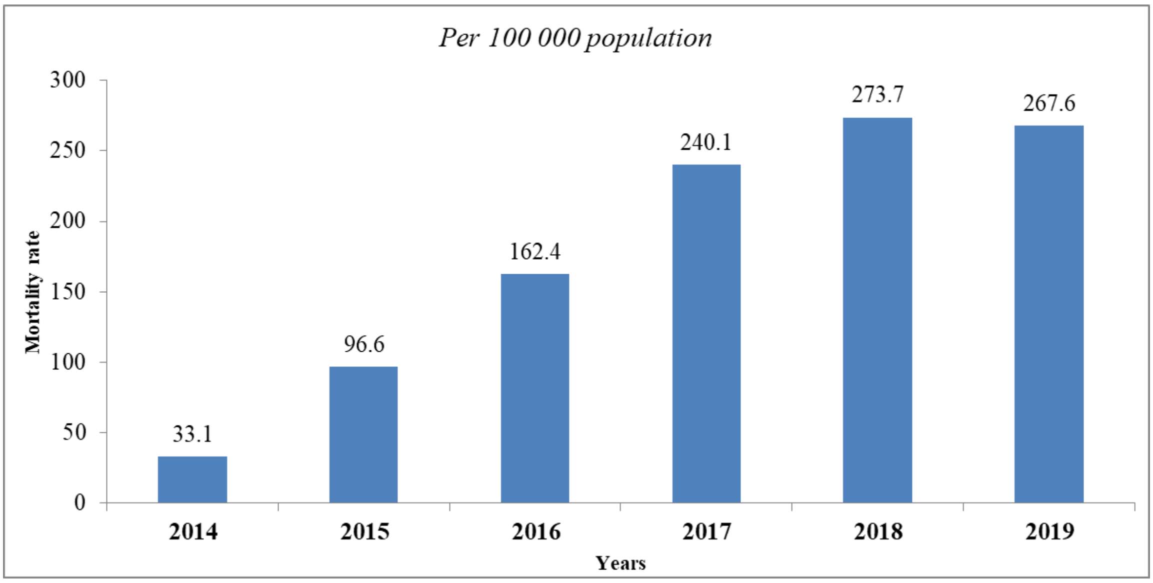

B.

Table S1. *Multivariable Cox proportional hazards regression analysis with unadjusted and adjusted values for 2014 – 2019 years.*

**Variables**	**Unadjusted (95% CI)**	**p-value**	**Model 1* (95% CI)**	**p-value**	**Model 2** (95% CI)**	**p-value**
**Gender**						
Women	Ref.		Ref.		Ref.	
Men	1.71 [1.69 - 1.72]	*p<0.001*	1.98 [1.96 - 2.00]	*p<0.001*	1.93 [1.91 - 1.95]	*p<0.001*
**Age groups**						
18-24	Ref.		Ref.			
25-34	1.41 [1.09 - 1.81]		1.59 [1.23 - 2.05]		1.49 [1.15 - 1.94]	
35-44	1.95 [1.54 - 2.49]		2.29 [1.81 - 2.92]		2.19 [1.72 - 2.81]	
45-54	3.13 [2.46 - 3.97]	*p<0.001*	3.73 [2.94 - 4.73]	*p<0.001*	3.52 [2.75 - 4.49]	*p<0.001*
55-64	6.34 [4.99 - 8.04]		7.59 [5.98 - 9.64]		6.97 [5.46 - 8.89]	
65-74	13.38 [10.54 - 16.98]		16.52 [13.01 - 20.97]		14.86 [11.64 - 18.97]	
75<	35.68 [28.11 - 45.31]		45.66 [35.97 - 57.97]		40.44 [31.68 - 51.62]	
**Ethnicity**						
Kazakh	Ref.		Ref.			
Russian	1.56 [1.54 - 1.58]	*p<0.001*	1.18 [1.17 - 1.19]	*p<0.001*	1.21 [1.19 - 1.22]	*p<0.001*
Other	1.43 [1.41 - 1.45]		1.15 [1.13 - 1.17]		1.15 [1.14 - 1.17]	
**Place of residence**						
Urban	Ref.		Ref.			
Rural	0.88 [0.88 - 0.89]	*p<0.001*	1.06 [1.05 - 1.07]	*p<0.001*	1.14 [1.13 - 1.15]	*p<0.001*
**Comorbidities**						
Diabetes	1.32 [1.30 - 1.34]		1.35 [1.33 - 1.36]		1.27 [1.25 - 1.29]	
Stroke	3.31 [3.26 - 3.36]	*p<0.001*	2.69 [2.65 - 2.73]	*p<0.001*	2.62 [2.58 - 2.66]	*p<0.001*
Chronic Kidney Disease	3.49 [3.35 - 3.65]		4.54 [4.35 - 4.74]		4.18 [3.99 - 4.36]	

**Model 1: adjusted for sex and age.*

***Model 2: adjusted for Model1+ethnicity, residence and comorbidities.*
